# Isolation and identification of induced systemic resistance determinants from *Bacillus simplex* Sneb545 against *Heterodera glycines*

**DOI:** 10.1038/s41598-020-68548-4

**Published:** 2020-07-14

**Authors:** Zhifu Xing, Xiaojing Wu, Jing Zhao, Xuebing Zhao, Xiaofeng Zhu, Yuanyuan Wang, Haiyan Fan, Lijie Chen, Xiaoyu Liu, Yuxi Duan

**Affiliations:** 1 College of Plant Protection, Shenyang Agricultural University, Shenyang, Liaoning China; 2 College of Biology Science and Technology, Shenyang Agricultural University, Shenyang, Liaoning China; 3 College of Science, Shenyang Agricultural University, Shenyang, Liaoning China

**Keywords:** Plant immunology, Bacterial techniques and applications

## Abstract

*Heterodera glycines* is one of the most destructive pathogens of soybean. Soybean seeds coated with *Bacillus simplex* Sneb545 have shown resistance to *H. glycines* as a result of induced systemic resistance (ISR) in the plants. In this study, we aimed to identify the resistance-inducing determinants from this *B. simplex* strain. Combining the ISR bioassay, six ISR-active compounds were isolated from a culture of *B. simplex* Sneb545 using organic solvent gradient extraction, silica gel column chromatography, Sephadex LH-20 column chromatography, and semi-preparative high-performance liquid chromatography (HPLC), and all systems were based on activity tracking. The compounds were determined as cyclic(Pro-Tyr), cyclic(Val-Pro), cyclic(Leu-Pro), uracil, phenylalanine, and tryptophan using ^1^H NMR and ^13^C NMR. In plants from seeds coated with *Bacillus simplex* Sneb545, these six ISR-active compounds delayed the development of *H. glycines* in soybean roots. Moreover, cyclic(Pro-Tyr), cyclic(Val-Pro), and tryptophan reduced the number of nematodes in soybean roots. The expression levels of defense-related genes with cyclic(Val-Pro), tryptophan and uracil treatment soybean analysed using Quantitative real-time PCR (qRT-PCR). The results indicate cyclic(Val-Pro), tryptophan and uracil induced the expression of defense-related genes involved in the SA- and JA-pathways to against *H. glycines*. Our research results provide new agents for the control of *H. glycines*.

## Introduction

Soybean is an important economic crop, which is widely used in oil and food production. The United States of America, Brazil, and Argentina are the top three producers of soybean, producing approximately 123.7, 117.0, and 55.0 million metric tons annually, respectively, China is ranked fourth globally, with a production of 15.9 million metric tons per year (https://soystats.com/international-world-soybean-production, 2019). *Heterodera glycines* (soybean cyst nematode, SCN) is one of the most destructive pathogens of soybean^1^. This nematode is a considerable threat to soybean production and is responsible for annual losses of up to USD 1.5 billion globally^2^. Chemical nematicides are widely used to control parasitic nematode disease in soybean^3^. However, their application not only causes widespread environmental pollution but also threatens human health. In addition, drug tolerance of nematodes has been enhanced by the long-term use of chemical nematicides^4,5^. It is necessary, therefore, to seek safe, effective, and environmentally friendly products to defend soybean plants against SCN^6^.

The life cycle of SCN consists of four juvenile stages (J1–J4). The embryo in the egg develops into the first stage (J1) of juvenile and then molts in the egg to form the infested second-stage juveniles (J2). The J2 penetrates the root of the host plant, once J2 reach the vascular cylinder, they will establish a feeding site at the root, and then begins to develop into the third (J3) and fourth (J4) juvenile stages in the root system. Finally, matures to an adult female or male and most become adults by 30 days post-infection^7,8^.

Plants have evolved a great variety of defence mechanisms to fight against pathogens. Plant growth-promoting rhizobacteria (PGPR) play an important role in plant defence by triggering induced systemic resistance (ISR)^9,10^. The exogenous stimulation of PGPR can induce a plant immune response, which can directly induce salicylic acid (SA)- and/or jasmonic acid (JA)-dependent defence responses^11,12^. Previous studies have indicated that the expression of SA- and JA-pathway-related genes could be up-regulated in host plants in response to SCN by coating seeds with bacteria^13–15^.

Secondary metabolites produced by PGPR, identified as ISR determinants, play a role in resisting plant pathogens by triggering the ISR response^16–18^. ISR determinants, such as flagellin, pyoverdine, and N-alkylated benzylamine of *Pseudomonas* spp., affect the level of phytohormones and enhance resistance against disease by regulating the physiological and metabolic responses of plants^19–23^. In response to ISR, SA in *Pseudomonas aeruginosa* enhanced plant resistance to *Meloidogyne javanica* and promoted the colonization of bacteria into the tomato rhizosphere^24^. In addition, tomato seeds soaked with an SA-chitosan mixture improved phenylalanine ammonia lyase (PAL) activity and SA content, which increased the resistance of tomato plants infected with nematodes^25,26^. Lipopolysaccharide (LPS) of *Rhizobacterium rhizobium* protects the potato from *Globodera pallida*^27^, and N-acyl-L-homoserine lactone (AHL) plays a key role in the biocontrol activity of rhizobacteria against *Alternaria alternata*^28^. Additionally, the phthalic acid methyl ester of *Bacillus subtilis* acts against *Fusarium* wilt in tomato^29^. Therefore, understanding the roles of such secondary metabolites can inform the management of plant-parasitic nematodes.

It is important to explore the basic mode of function for improving the effectiveness of biocontrol agents. Studies have shown that soybean seeds coated with *Bacillus simplex* Sneb545 showed reduced infection and delayed development of pathogenic nematodes by triggering ISR^30,31^. This study was aimed to isolate and identify the ISR determinants from *Bacillus simplex* Sneb545 against *H. glycines*. Six isolated ISR-active compounds were obtained through organic solvent gradient extraction, silica gel column chromatography, Sephadex LH-20 column chromatography, and semi-preparative high-performance liquid chromatography (HPLC). We also analysed the expression levels of defense-related genes involved in SA and JA pathway of soybean treated with cyclic(Val-Pro), tryptophan and uracil treatment soybean by using qRT-PCR. Our research results provide new agents for the control of *H. glycines*.

## Results

### Preliminary screening of ISR determinants from *Bacillus simplex* Sneb545

**Figure 1:**
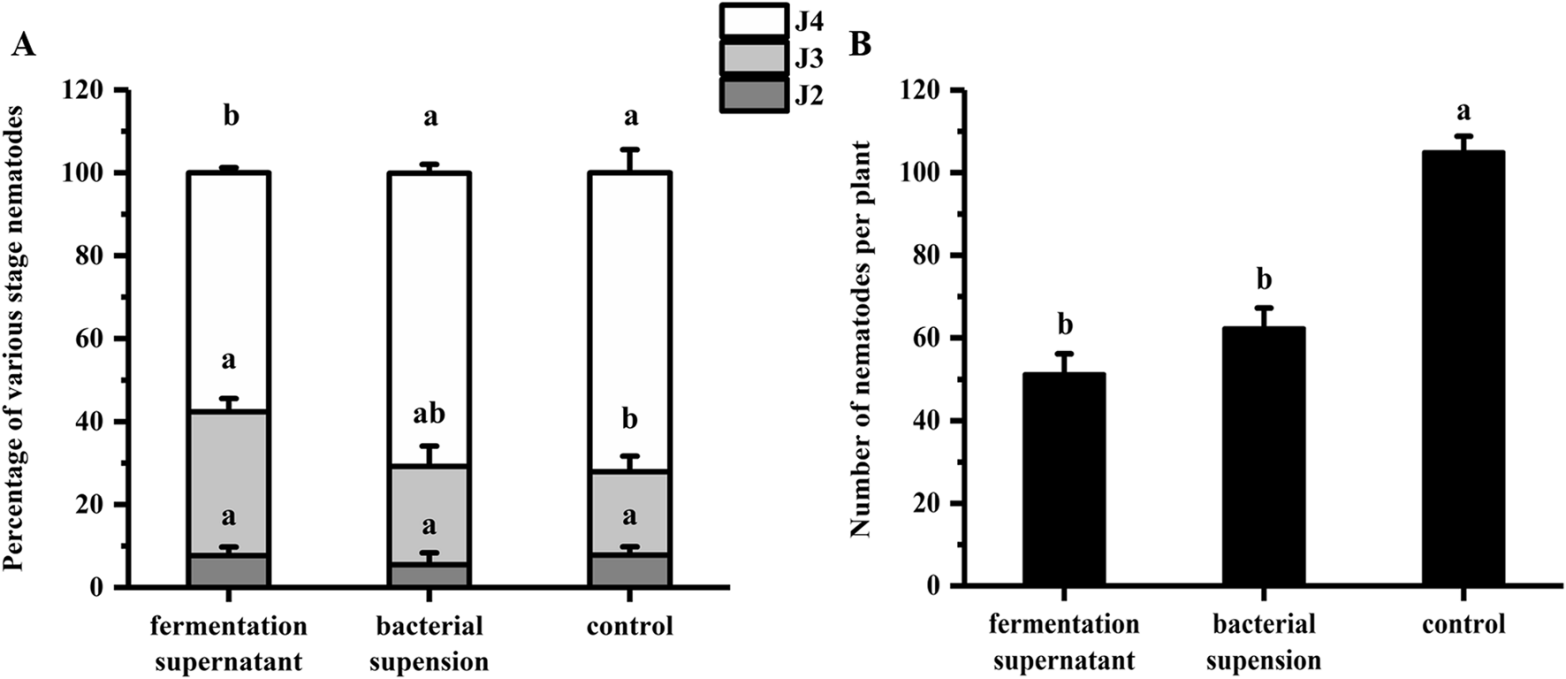
Primary screening of ISR determinants from *B. simplex* Sneb545. Soybean seeds treated with fermentation supernatant, bacterial suspension, and a control (sterile water) at 12 dpi inoculation. (**A**) Percentages of various stage nematodes. The ratio of different developmental stages of SCN to the total number of nematodes within soybean roots. (**B**) Number of nematodes per plant. The different lowercase letters on the bars represent significant differences (*P* < 0.05) between treatments. Data are means ± SE. n = 3.

Soybean seeds were coated with fermentation supernatant and bacterial suspension for ISR bioassay. The results indicate that the J4 ratio (the numbers of J4 to the total nematodes in per plant root) was 59.8% in the fermentation supernatant treatment, which was significantly lower than the 71.1% in the bacterial suspension treatment and the 72.1% in the control (Fig. 1A). The numbers of nematodes in the root were 51 and 62 in the fermentation supernatant and bacterial suspension, respectively, which were significantly lower than that of the control (Fig. 1B). Thus, the fermentation supernatant was used for further separation and purification that considered by combining the J4 ratio and total number nematodes in the root system.

### Extraction of ISR determinants from *Bacillus simplex* Sneb545 supernatant

**Figure 2:**
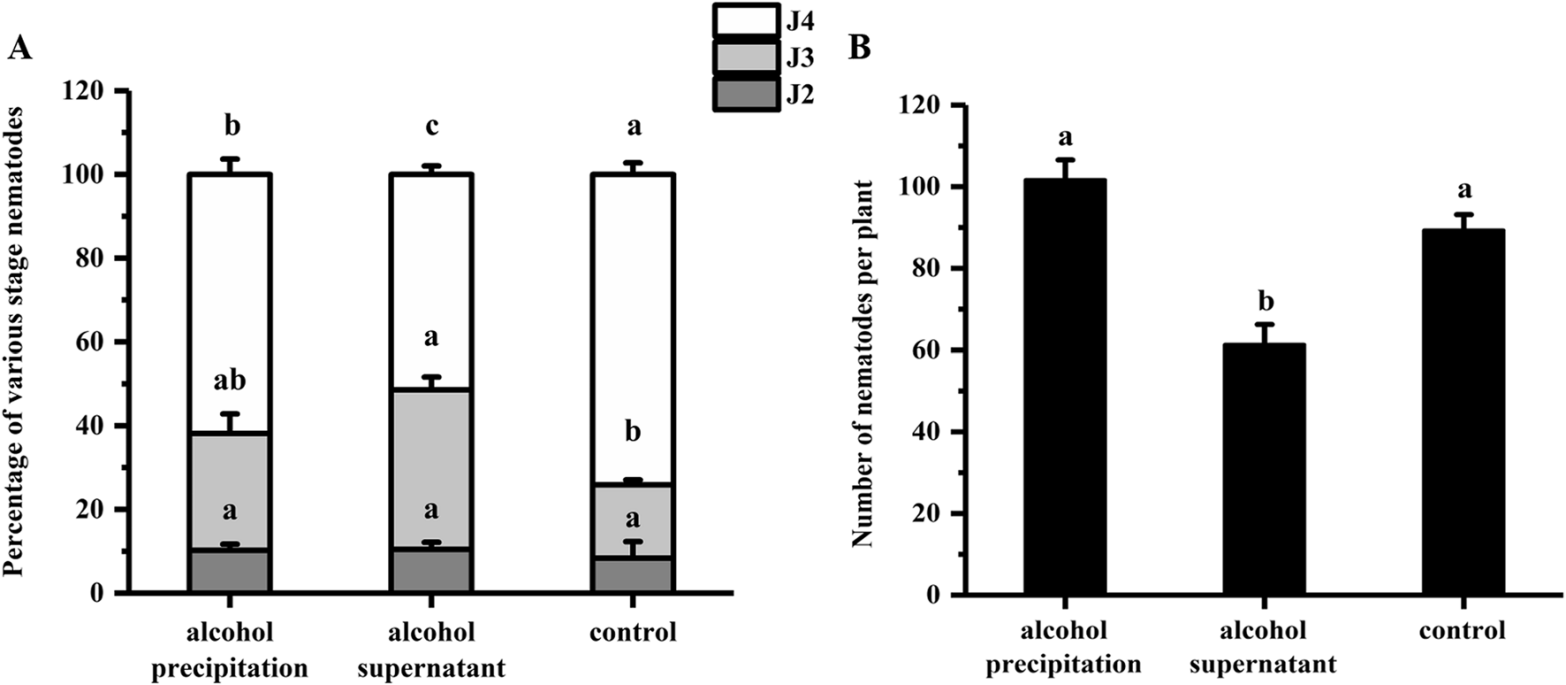
ISR bioassay for alcohol precipitate and alcohol supernatant. The soybean seeds treated with alcohol precipitate, alcohol supernatant, and control (2% DMSO) at 12 dpi inoculation. (**A**) Percentage of various stage nematodes. The ratio of different developmental stages of SCN to the total number of nematodes within soybean roots. (**B**) Number of nematodes per plant. The different lowercase letters on the bars represent significant differences (*P* < 0.05) between treatments. The data are means ± SE. n = 3.

Soybean seeds were coated with alcohol supernatant and precipitate for ISR bioassay. The results indicate that the J4 ratio was 51.6% in the alcohol supernatant treatments, which was significantly lower than the 74.4% in the control (Fig. 2A). There were 61 nematodes in the roots of the alcohol supernatant treatment plants, which was significantly lower than the number in the control and the ethanol precipitate treatments (Fig. 2B). These findings indicate that the potential ISR determinants were in the alcohol supernatant, therefore, the alcohol supernatant was used for further separation and purification.

**Figure 3:**
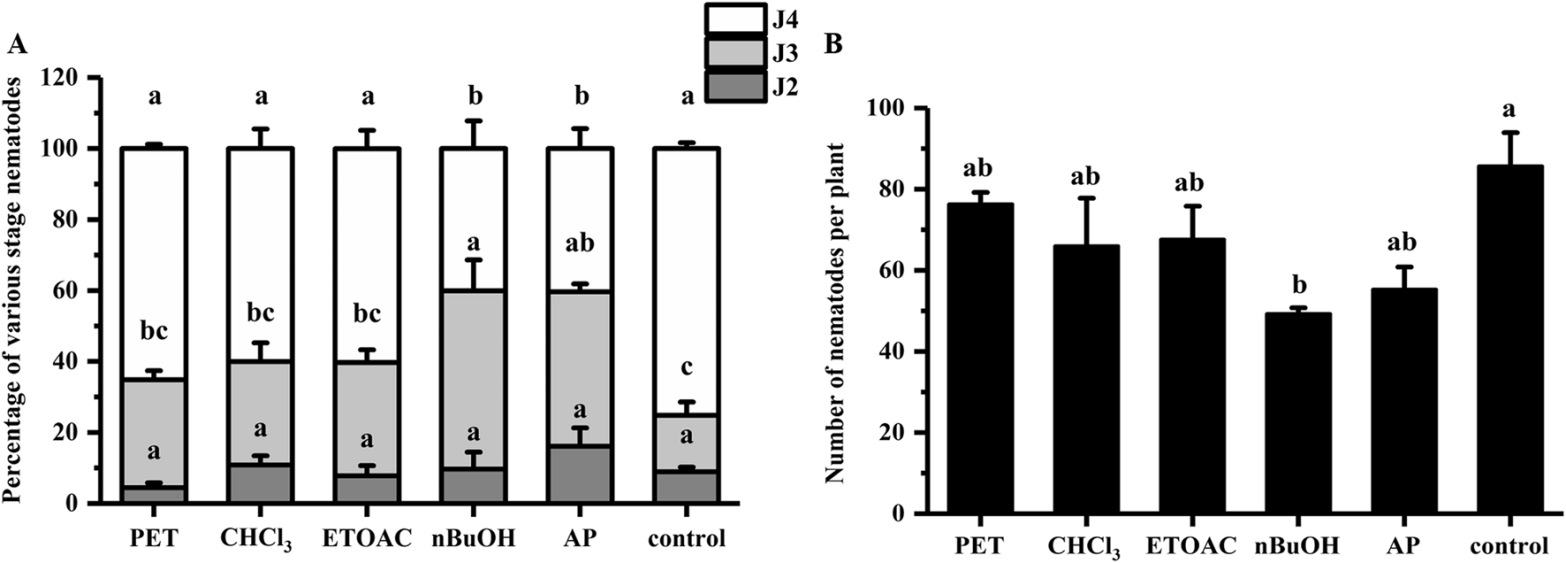
ISR bioassay for various organic extracts. The soybean seeds treated with PET, CHCl_3_, ETOAC, nBuOH, AP, and control (2%DMSO) at 12 dpi inoculation. (**A**) Percentage of various stage nematodes. The ratio of different developmental stages of SCN to the total number of nematodes within soybean roots. (**B**) Number of nematodes per plant. The different lowercase letters on the bars represent significant differences (*P* < 0.05) between treatments. The data are means ± SE. n = 3.

The alcohol supernatant solution was extracted using a series of organic solvents, and ISR bioassay was performed using the final aqueous phase (AP) and various organic extracts. The J4 ratio was 65.1%, 58.9%, 60.1%, 39.9%, and 40.4% for the petroleum ether (PET), chloroform (CHCl_3_), ethyl acetate (ETOAC), n-butanol (nBuOH) and final AP treatments, respectively (Fig. 3A), while the control was 73.8%; the number of nematodes in the roots were 76, 66, 67, 49, and 55, respectively (Fig. 3B). The values for the nBuOH treatments were significantly lower than those of the control, indicating that the ISR-active components were retained in the nBuOH fraction, which was further separated and purified.

### Separation and purification of n-butanol extract

**Figure 4:**
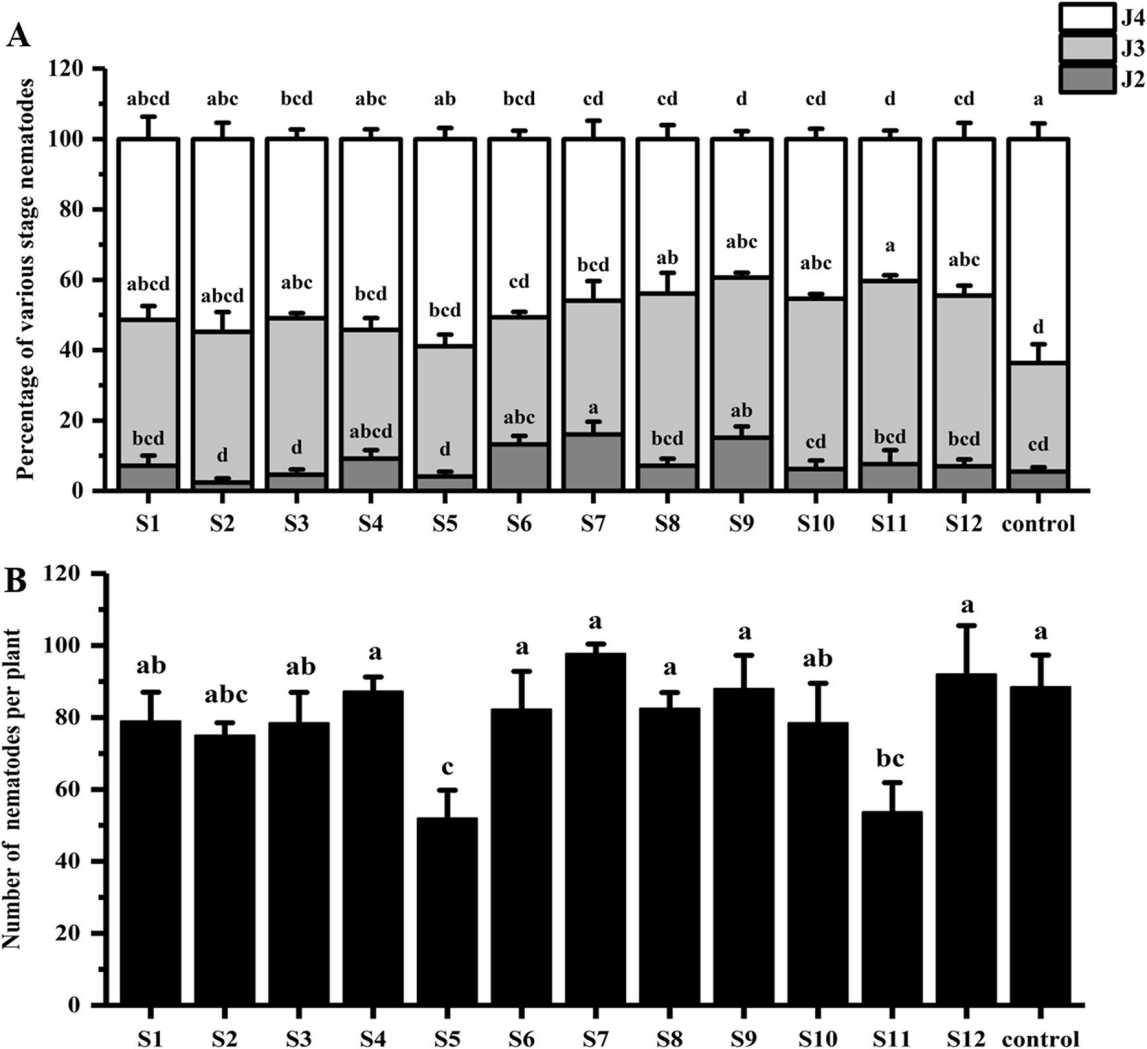
ISR bioassay for various nBuOH extract components. The soybean seeds treated with 12 fractions (S1−S12) extracted from nBuOH and the control (2%DMSO) at 12 dpi inoculation. (**A**) Percentage of various stage nematodes. The ratio of different developmental stages of SCN to the total number of nematodes within soybean roots. (**B**) Number of nematodes per plant. The different lowercase letters on the bars represent significant differences (*P* < 0.05) between treatments. The data are means ± SE. n = 3.

Twelve fractions (S1−S12) were obtained from the n-butanol extract by combining silica gel chromatography and thin layer chromatography and these were then used for ISR bioassays. The J4 ratio for S5 and S11 within the root was 58.7% and 40.9%, respectively (Fig. 4A). The number of nematodes in the roots was 52 and 54 (Fig. 4B) for S5 and S11, respectively, which were significantly lower than the control. Combined with the J4 ratio and number of nematodes in the roots, the S5 and S11 were further separations.

### Separation fraction S5 through semi-preparative HPLC

**Figure 5:**
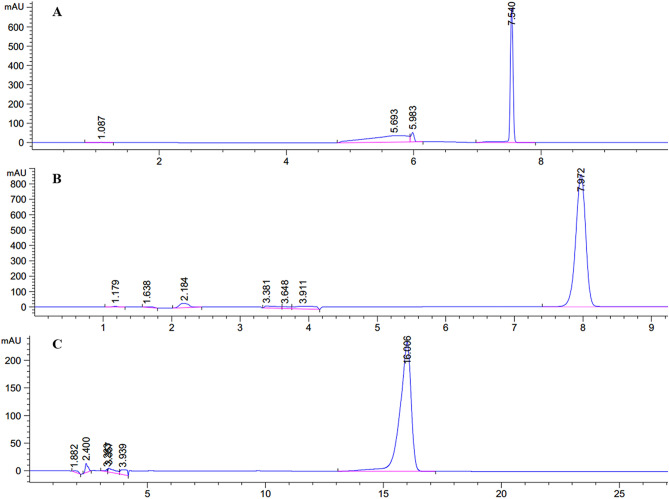
Separation of S5 by HPLC. (**A**) HPLC spectrum of S5-1; (**B**) HPLC spectrum of S5-2; (**C**) HPLC spectrum of S5-3.

Three main compounds were obtained in fraction S5 by using semi-preparative HPLC. The sub-fractions S5-1, S5-2, and S5-3 were the peak points at 7.540 min, 7.972 min, and 16.006 min, respectively (Fig. 5A,B,C).

### Separation of S11 by Sephadex LH-20 column

**Figure 6:**
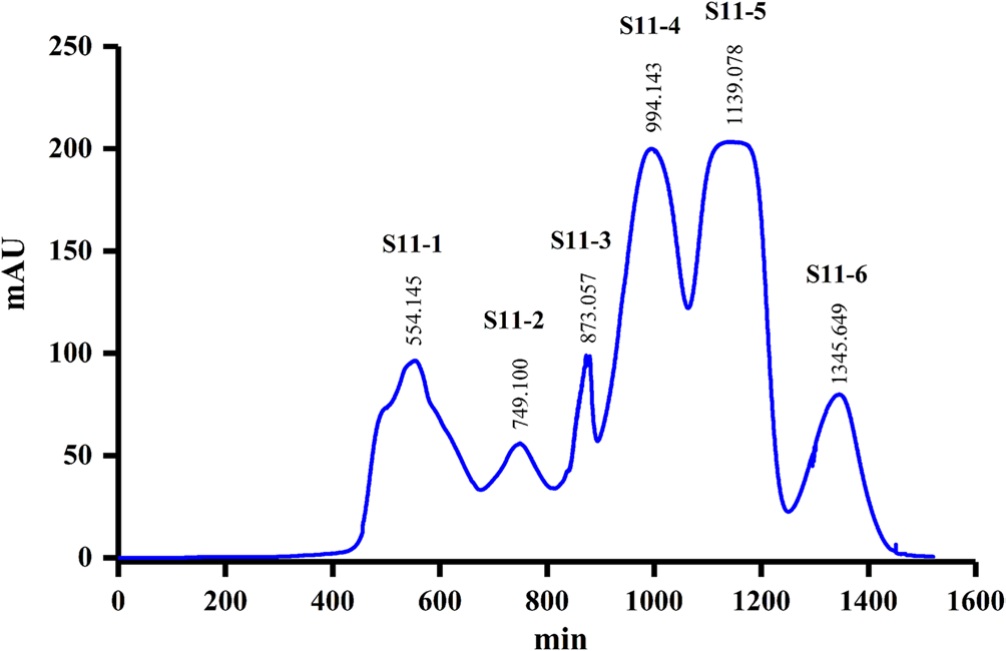
Separation of S11 by Sephadex LH-20 column chromatography. The ultraviolet detector determined S11 on the 220 nm and obtained S11-1, S11-2, S11-3, S11-4, S11-5, and S11-6 sub-fractions.

**Figure 7:**
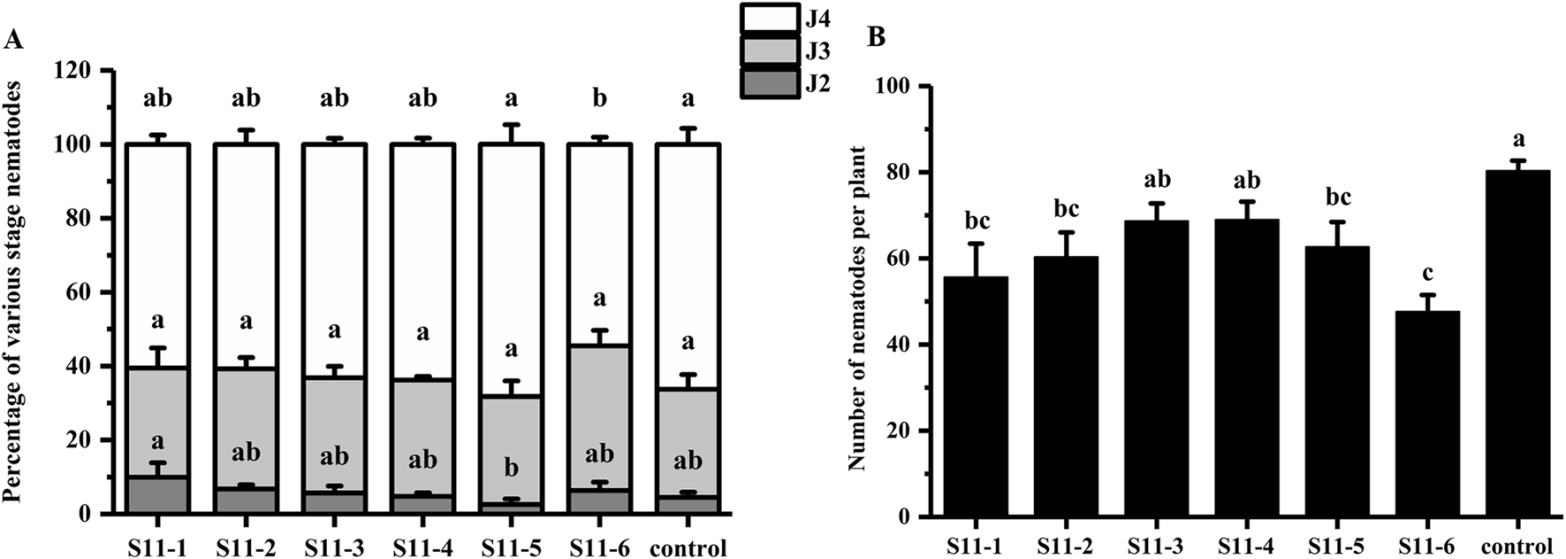
ISR bioassay for the sub-fraction of S11 by Sephadex LH-20 column chromatography. The soybean seeds treated with S11-1, S11-2, S11-3, S11-4, S11-5, S11-6 sub-fraction and control (2%DMSO) at 12 dpi inoculation. (**A**) Percentage of various stage nematodes. The ratio of different developmental stages of SCN to the total number of nematodes within soybean roots. (**B**) Number of nematodes per plant. The different lowercase letters on the bars represent significant differences (*P* < 0.05) between treatments. The data are means ± SE. n = 3.

Fraction S11 was further purified using a Sephadex LH-20 column and the ultraviolet detector (220 nm) determined six sub-fractions named S11-1, S11-2, S11-3, S11-4, S11-5, and S11-6 (Fig. 6). The six sub-fractions were subjected to ISR bioassays. The J4 ratio of S11-6 was 54.5% (Fig. 7A) and the number of nematodes in the roots was 48 (Fig. 7B) for S11-6, which were significantly lower than the control. The results suggest that S11-6 exhibited ISR-activity.

### Separation of S11-6 by semi-preparative HPLC

**Figure 8:**
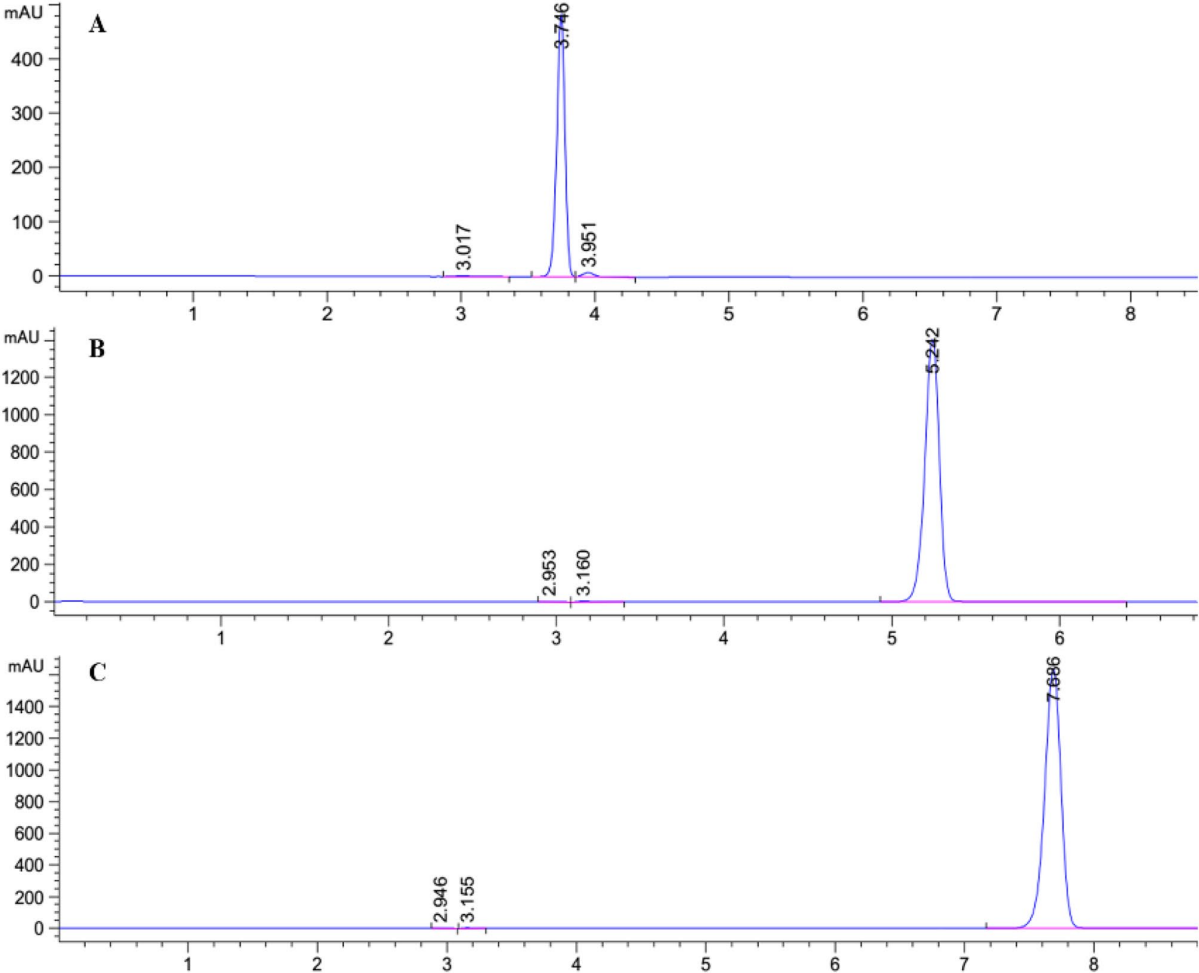
Separation of S11-6 components by HPLC. (**A**) HPLC spectrum of S11-6-1; (**B**) HPLC spectrum of S11-6-2; **C** HPLC spectrum of S11-6-3.

Three main compounds were found in the fraction S11-6 by using semi-preparative HPLC. The sub-fractions S11-6-1, S11-6-2, and S11-6-3 were the peak points at 3.745 min, 5.242 min, and 7.686 min, respectively (Fig. 8A,B,C).

### Identification of active compounds

**Figure 9:**
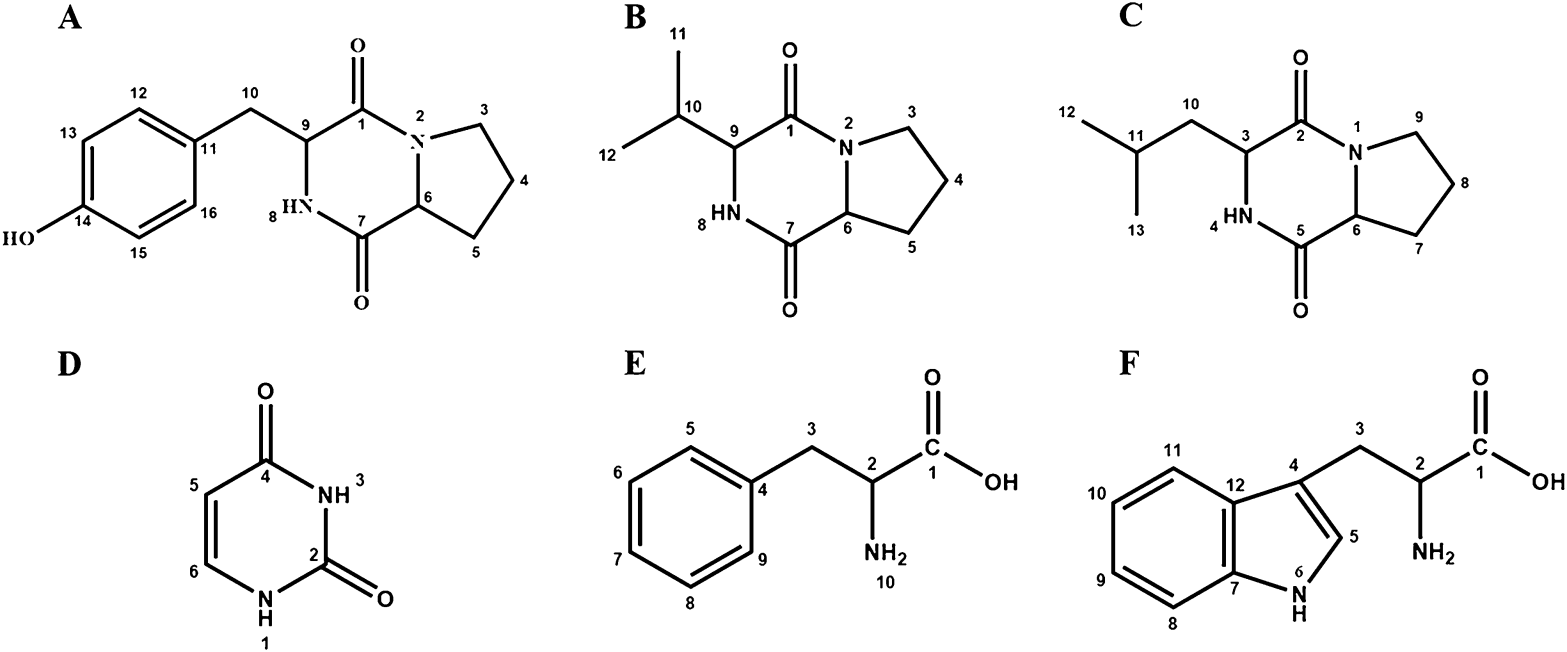
Structural formulae of six compounds. (**A**) cyclic(Pro-Tyr); (**B**) cyclic(Val-Pro); (**C**) cyclic(Leu-Pro); (**D**) uracil; (**E**) phenylalanine; (**F**) tryptophan.

Compound S5-1: white powder. ^1^H NMR (600 MHz, DMSO-*d*6) δ: 9.18 (1H, s, OH), 7.86 (1H, s, NH), 7.05 (2H, m, H-12, H-16), 6.64 (2H, m, H-13, H-15), 4.24 (1H, t, H-9), 4.02 (1H, d, H-6), 3.40 (1H, m, H-3a), 3.26 (1H, m, H-3b), 2.00 (1H, m, H-5a), 1.73 (1H, m, H-5b), 1.84 (2H, m, H-4). ^13^C NMR (600 MHz, DMSO-*d*6) δ: 169.25 (C-7), 165.45 (C-1), 156.23 (C-14), 131.16 (C-12, 16), 127.41 (C-11), 115.12 (C-13, 15), 58.74 (C-6), 56.36 (C-9), 44.90 (C-3), 35.05 (C-10), 28.06 (C-5), 22.21 (C-4). According to the literature^32^, the compound was identified as cyclic(Pro-Tyr) (Fig. 9A).

Compound S5-2: white powder. ^1^H NMR (600 MHz, DMSO-*d*6) δ: 7.96 (1H, s, NH), 4.12 (1H, t, H-6), 3.92 (1H, ddd, H-9), 3.43(1H, m, H-3a), 3.40 (1H, dt, H-3b), 2.34 (1H, m, H-5a), 2.13 (1H, m, H-5b), 1.82 (2H, m, H-4), 1.19 (1H, dq, H-10), 1.01 (3H, d, H-11), 0.85 (3H, d, H-12). ^13^C NMR (600 MHz, DMSO-*d*6) δ: 170.65 (C-1), 165.61 (C-7), 59.84 (C-6), 58.62 (C-9), 45.01 (C-3), 28.23 (C-10), 28.19 (C-5), 22.46 (C-4), 18.71 (C-11), 16.76 (C-12). According to the literature^32^, the compound was identified as cyclic(Val-Pro) (Fig. 9B).

Compound S5-3: white powder. ^1^H NMR (600 MHz, CD_3_OD) δ: 7.93(1H, s, NH), 4.28 (1H, t, H-6), 4.15 (1H, m, H-3), 3.53 (2H, m, H-9), 2.32 (1H, m, H-7a), 2.04 to 1.92 (5H, m, H-7b, H-8, H-10), 1.54 (1H, m, H-11), 0.98 (6H, m, H-12, H-13). ^13^C NMR (600 MHz, CD_3_OD) δ: 171.83 (C-2), 167.47 (C-5), 8.75 (C-6), 53.14 (C-3), 44.95 (C-9), 37.91 (C -10), 27.58 (C-7), 24.27 (C-11), 22.16 (C-8), 21.80 (C-12 or C-13), 20.71 (C-12 or C-13). According to the literature^33^, the compound was identified as cyclic(Leu-Pro) (Fig. 9C).

Compound S11-6-1: white powder. ^1^H NMR (600 MHz, DMSO-*d*6) δ: 10.93 (1H, s, NH), 10.79 (1H, s, NH), 7.42 (1H, d, H-6), 5.47 (1H, d, H-5). ^13^C NMR (600 MHz, DMSO-*d*6) δ: 164.74 (C-4), 154.5 (C-2), 142.2 (C-6), 100.3 (C-5). According to the literature^34^, the compound was identified as uracil (Fig. 9D).

Compound S11-6-2: white powder. ^1^H NMR (600 MHz, D_2_O) δ: 7.42 (2H, m, H-11, 13), 7.37 (1H, t, H-12), 7.32 (2H, m, H-10, 14), 3.99 (1H), dd, H-2), 3.28 (1H, dd, H-3a), 3.12 (1H, dd, H-3b). ^13^C NMR (600 MHz, D_2_O) δ: 173.80 (C-1), 134.96 (C-4), 127.57 to 129.24 (C5-9), 55.91 (C-2), 36.22 (C-3). According to the literature^35^, the compound was identified as phenylalanine (Fig. 9E).

Compound S11-6-3: white powder. ^1^H NMR (600 MHz, D_2_O) δ: 10.20 (1H, d, NH-6), 7.73 (1H, d, H-11), 7.53 (1H, d, H-8), 7.31 (1H, s, H-5), 7.28 (1H, ddd, H-10), 7.20 (1H, td, H-9), 4.04 (1H, dd, H-2), 3.48 (1H, dd, H-3a), 3.30 (1H, dd, H-3b). ^13^C NMR (600 MHz, D_2_O) δ: 174.27 (C-1), 136.20 (C-7), 126.51 (C-12), 124.85 (C-5), 121.96 to 111.77 (C8-11), 107.33 (C-4), 54.92 (C-2), 26.24 (C-3). According to the literature^36^, the compound was identified as tryptophan (Fig. 9F).

### Active compounds from ISR bioassay

**Figure 10:**
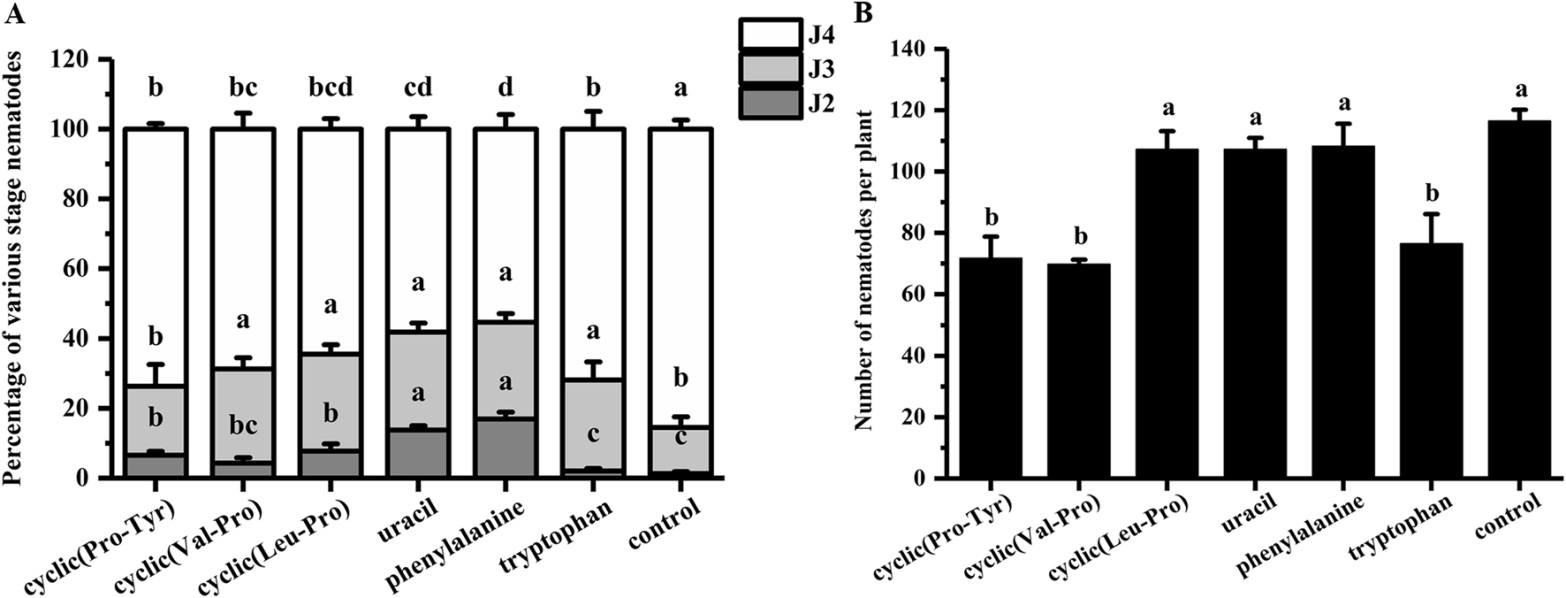
ISR bioassay for six active compounds. The soybean seeds treated with cyclic(Pro-Tyr), cyclic(Val-Pro), cyclic(Leu-Pro), uracil, phenylalanine, and tryptophan and a control (2%DMSO) at 12 dpi inoculation. (**A**) Percentage of various stage nematodes. The ratio of different developmental stages of SCN to the total number of nematodes within soybean roots. (**B**) Number of nematodes per plant. The different lowercase letters on the bars represent significant differences (*P* < 0.05) between treatments. The data are means ± SE. n = 3.

The obtained active compounds, cyclic(Pro-Tyr), cyclic(Val-Pro), cyclic(Leu-Pro), uracil, phenylalanine, and tryptophan, were used for ISR bioassays. The J4 ratio was 64.5%, 58.2%, and 55.2% for cyclic(Leu-Pro), uracil, and phenylalanine, respectively, which were significantly lower than that of the control (85.6%; Fig. 10A). The number of nematodes in the roots was not significantly different from that of the control (Fig. 10B). The results indicated that cyclic(Leu-Pro)-, uracil-, and phenylalanine-coated soybean seeds delayed the development of nematodes in the plant roots.

The numbers of nematodes in the roots were 71, 69, and 76 (Fig. 10B) for cyclic(Pro-Tyr), cyclic(Val-Pro), and tryptophan, respectively, which were significantly lower than that of the control (117). Moreover, the J4 ratio of cyclic(Pro-Tyr), cyclic(Val-Pro), and tryptophan were 73.7%, 68.7%, and 71.9%, which were significantly lower than the 85.6% for the control (Fig. 10A). Therefore, cyclic(Pro-Tyr)-, cyclic(Val-Pro)-, and tryptophan-coated soybean seeds had a significant effect on the number and development of nematodes in roots.

### Induced defense-related gene expression in the root

**Figure 11:**
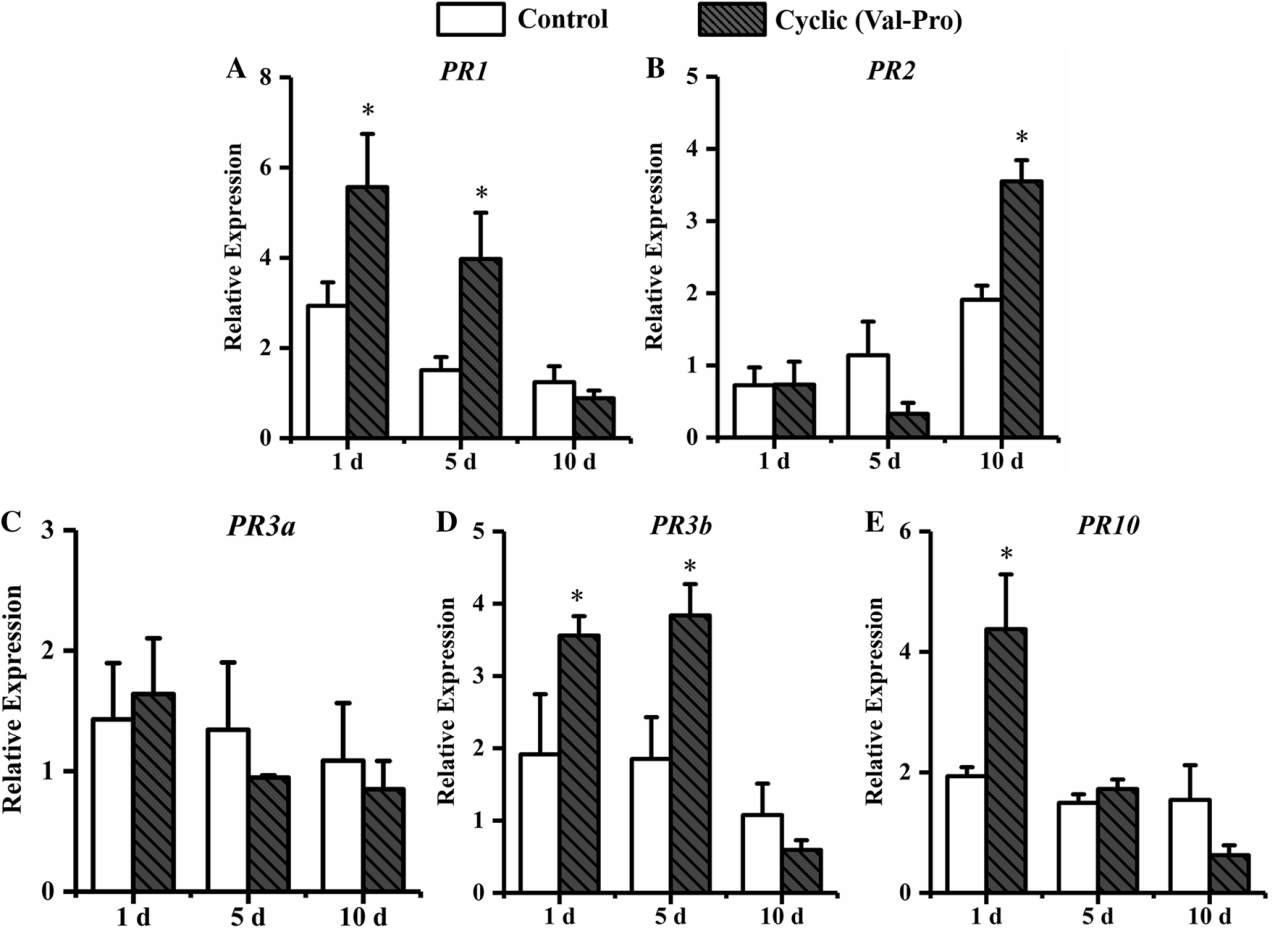
The expression of defense genes in soybean roots with cyclic(Val-Pro) treatment. Soybean root samples were taken at 1, 5, and 10 d after *H. glycines* inoculation, and the expression levels of five defense genes were analyzed by qRT-PCR: (**A**) *PR1*, (**B**) *PR2*, (**C**) *PR3a*, (**D**) *PR3b* and (**E**) *PR10*. Error bars represent standard errors; a *t*-test was used to determine significant differences between the cyclic(Val-Pro)-treated sample and the control (∗ *P* < 0.05).

To test the active compounds mediated the ISR defense response against *H. glycine*, cyclic (Val-Pro), tryptophan and uracil were selected from six active compounds to treat soybeans, then the expression levels of SA-pathway-related genes (*PR1*, *PR2*) and JA-pathway-related genes (*PR3a*, *PR3b, PR10*) in soybeans were assessed by using qRT-PCR. Soybean was treated with cyclic (Val-Pro) and inoculated with *H. glycines* J2 (Fig. 11). During the stage of nematode infection, the expression of *PR1*, *PR3b* and *PR10* were significantly higher than that in control, which increasing 1.9-fold, 1.9-fold and 2.3-fold compared with that in control at 1 dpi. The transcript level of *PR1* and *PR3b* were 2.6-fold and 2.1-fold significantly higher than that in control at 5 dpi. A significantly enhanced transcription of *PR2* was 1.9-fold compared with that in control at 10 dpi.

**Figure 12:**
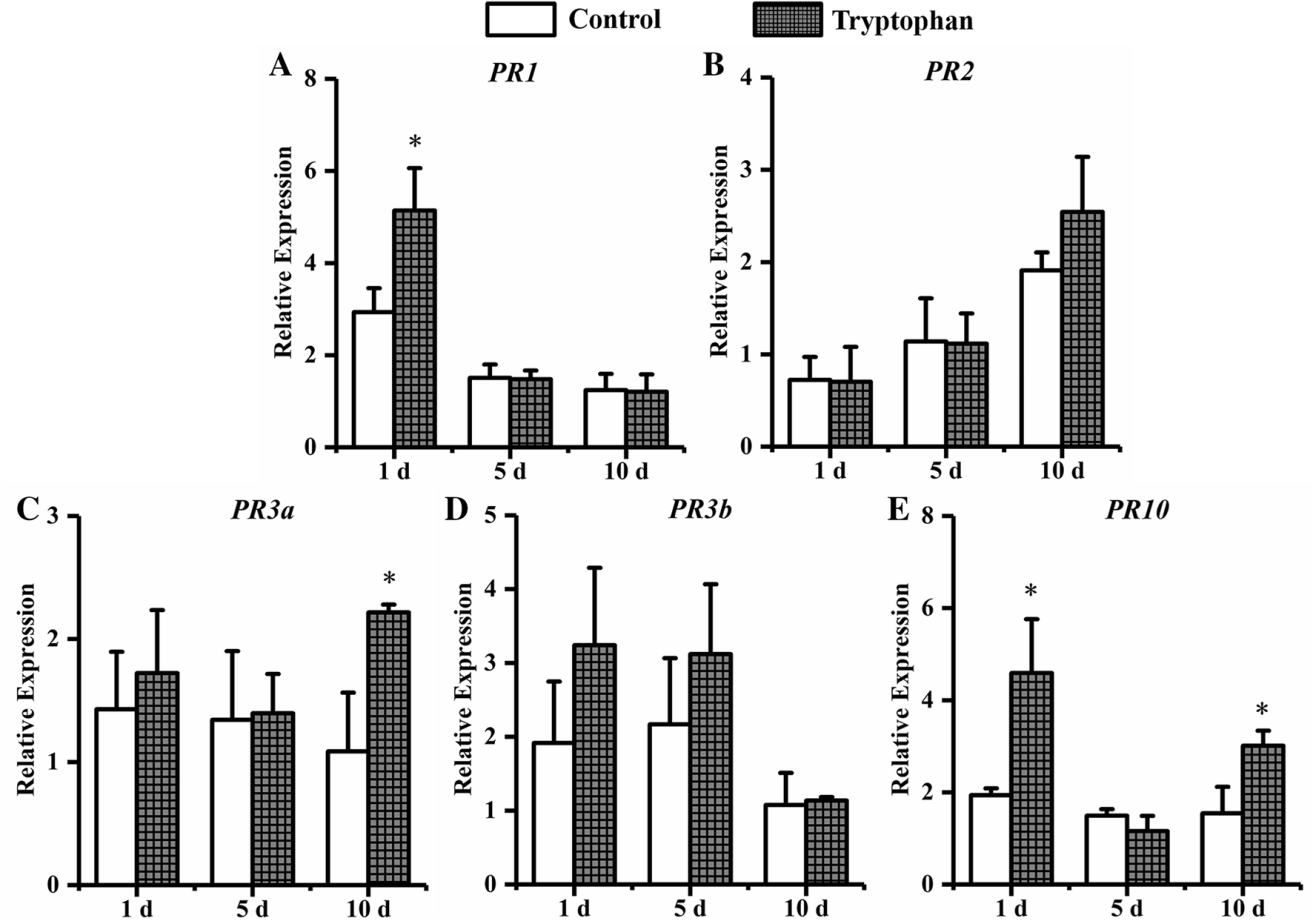
The expression of defense genes in soybean roots with tryptophan treatment. Soybean root samples were taken at 1, 5, and 10 d after *H. glycines* inoculation, and the expression levels of five defense genes were analyzed by qRT-PCR: (**A**) *PR1*, (**B**) *PR2*, (**C**) *PR3a*, (**D**) *PR3b* and (**E**) *PR10*. Error bars represent standard errors; a *t*-test was used to determine significant differences between the tryptophan-treated sample and the control (∗*P* < 0.05).

**Figure 13:**
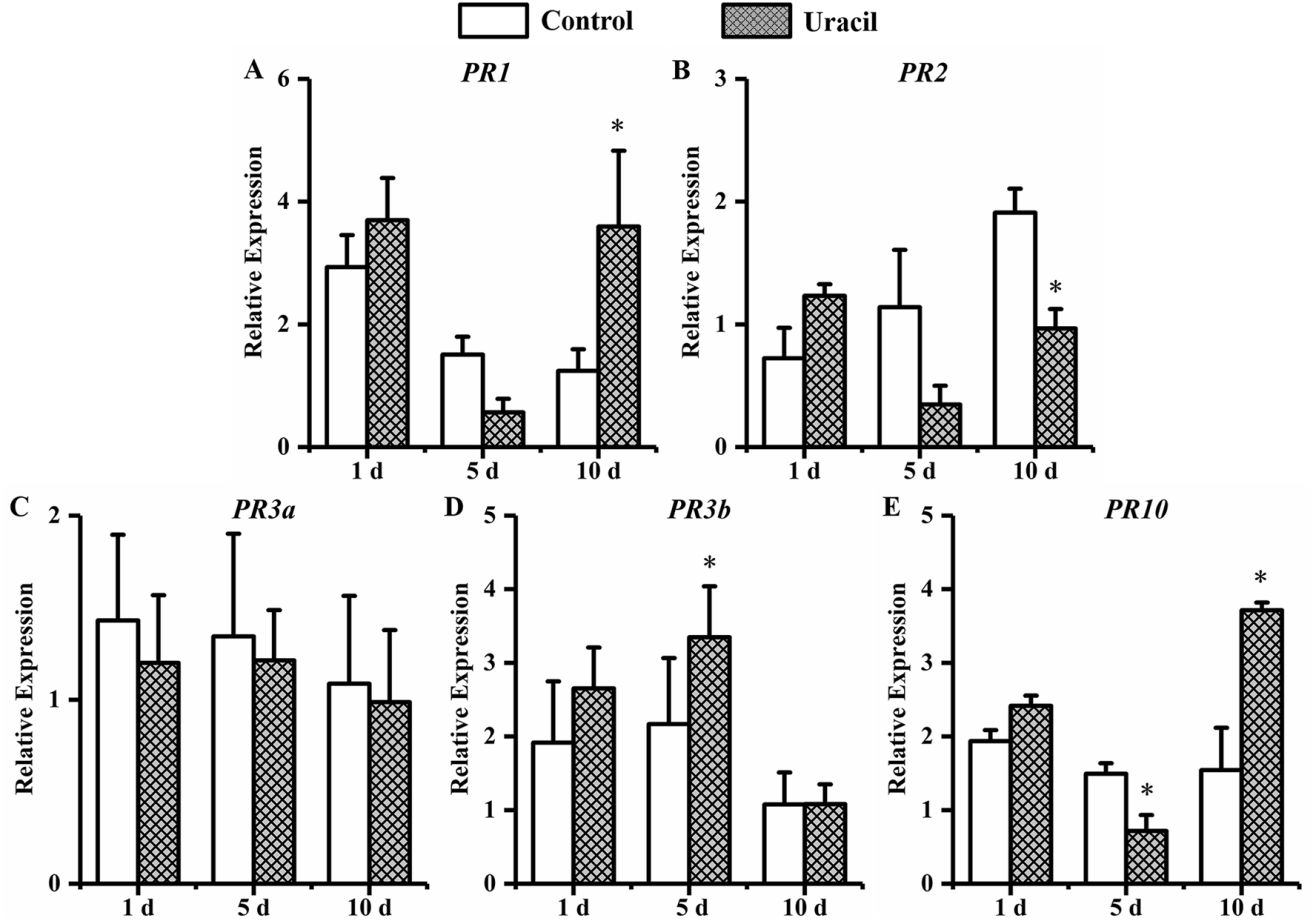
The expression of defense genes in soybean roots with uracil treatment. Soybean root samples were taken at 1, 5, and 10 d after *H. glycines* inoculation, and the expression levels of five defense genes were analyzed by qRT-PCR: (**A**) *PR1*, (**B**) *PR2*, (**C**) *PR3a*, (**D**) *PR3b* and (**E**) *PR10*. Error bars represent standard errors; a *t*-test was used to determine significant differences between the uracil-treated sample and the control (∗ *P* < 0.05).

Soybean was treated by tryptophan and inoculated *with H. glycines* (Fig. 12). At 1 dpi, the expression of *PR1* and *PR10* were significantly higher than that in control, which increasing 1.8-fold and 2.4-fold compared with that in control. The transcript level of *PR3a* and *PR10* were 2.0-fold and 1.9-fold significantly higher than that in control at 10 dpi. Soybean was treated by uracil and inoculated *with H. glycines* (Fig. 13). A significantly enhanced transcription of *PR3b* was 1.5-fold compared with that in control at 5 dpi. The transcript level of *PR1* and *PR10* were 2.9-fold and 2.4-fold significantly higher than that in control at 10 dpi.

## Discussion

Previous studies revealed that soybean seeds coasted with a fermentation broth of *B. simplex* Sneb545 showed reduced infestation and delayed development of nematodes as a result of ISR^30,31^. In this study, we assessed the ISR determinants by coating the soybean seeds with this bacterial strain, and all systems were based on activity tracking. Our results identified ISR determinants in the supernatant of the fermentation broth, which is consistent with the results of other studies^29,37^. Combining ISR bioassays, six ISR-activity compounds (S5-1, S5-2, S5-3, S11-6-1, S11-6-2, and S11-6-3) were obtained from the supernatant using progressive separation and purification steps. The compounds were finally identified as cyclic(Pro-Tyr), cyclic(Val-Pro), cyclic(Leu-Pro), uracil, phenylalanine, and tryptophan using the methods of ^1^H NMR and ^13^C NMR.

Cyclic(Pro-Tyr), cyclic(Val-Pro), and cyclic(Leu-Pro) are cyclic dipeptides that belong to 2,5-diketopiperazine (DKP) derivatives, which have a stable six-membered ring structure with two hydrogen bond donors and two hydrogen bond acceptors. Hydrogen bonds have an important effect on interactions between drugs and receptors, hence cyclic dipeptides play a key role in medicinal chemistry^38^. Cyclic(Ala-Val), cyclic(Pro-Tyr), and cyclic(Phe-Pro) are signal molecules that activate the biosensors of N-acyl-L-homoserine lactone (AHL), consequently regulating group behaviour^39^. In addition, natural cyclic dipeptides have a broad range of biological activities, such as antibacterial^40^, antifungal^41^, antiviral^42^, and antitumor^43^. Cyclic dipeptides from *B. vallismortis* induced resistance in *Arabidopsis* against *Pseudomonas* infection^44^. In this study, cyclic(Pro-Tyr), cyclic(Val-Pro), and cyclic(Leu-Pro) isolated from *B. simplex* against to *H. glycines* through ISR.

Uracil is a pyrimidine organic compound and exists in all living species. Pyrimidines play a substantial role in numerous aspects of plant metabolism and development^45^. Tomato seedlings were treated with uracil significantly increased the plant height and the fresh and dry weights of the shoot, and also resulted in the formation of more flowers^46^. Tobacco BY-2 cells regulate salvage activity of uracil to establishes proper cellular conditions for the induction of programmed cell death when increase s levels of nitric oxide and hydrogen peroxide^47^. In this study, uracil isolated from the *B. simplex* fermentation broth delayed the development of *H. glycines* in soybean root. Phenylalanine and tryptophan are amino acids that play a key role in plant growth and disease resistance^48^. Previous studies demonstrated that the contents of free amino acids increased in cotton seedlings inoculated with root-knot nematodes^49^. Phenylalanine has also been shown to suppress the development of nematodes in plants, and influences the survival of *M. javanica* J2^50^. Furthermore, phenylalanine is a precursor of SA biosynthesis. SA is a systemic signal for directing protein synthesis related to plant resistance to pathogens, which activates the defence responses of plant^51,52^. Tryptophan forms tryptamine through biosynthetic pathways, providing a common backbone for many secondary metabolites that may have different biological activities^53^, such as auxins (IAA), phytoalexins (camalexin), and alkaloids, which promote plant development and resist pathogen infection^54–56^. These results showed that the amino acids in plants play an important role in resistance to nematodes. In this study, phenylalanine and tryptophan isolated from the *B. simplex* fermentation broth delayed the development of *H. glycines*, which may have affected the amino acid content or regulated the metabolism level of phytoalexin in soybean.

SA and JA pathways have been implicated in plant resistance to pathogens. The PR proteins are involved in activating the defense response and are important in plant defenses against nematodes. According to the study reported that tomato plants pre-treated with SA and subsequently infected with root-knot nematodes, *PR1* was highly expressed in roots at 1 dpi^57^. During infection with *Phytophthora sojae*, the expression of *PR3b* greatly enhanced following the pre-treatment of soybean seedlings with benzothidiazole (BTH) in 24 h and the transcript abundance of *PR10* increased within 24 h following leaf pre-treatment with SA^58,59^. These results indicate that the SA- and JA-pathways play a role in pathogen infection. In this study, pre-treatment with cyclic(Val-Pro) significantly increased the expression of *PR1*, *PR3b*, and *PR10* and tryptophan increased the expression of *PR1* and *PR10* at 1 dpi. The results showed that the SA- and JA-pathways were involved in root resistance to nematode infection at an early stage. Another study reported that pre-treatment with *Pochonia chlamydosporia* induced the expression of the SA pathway (*PR1*) and JA pathway (*Lox D*) at 7 days after nematode inoculation^60^. In our study, high expression of *PR1*, *PR3b* in cyclic(Val-Pro) and *PR3b* in uracil were observed at 5 dpi. Similarly, the transcript levels of *PR2* in cyclic(Val-Pro) and *PR3a*, *PR10* in tryptophan, as well as *PR1*, *PR10* in uracil significantly increased at 10 dpi. This result suggests that the SA- and JA-pathways were involved in root resistance to nematode development. Overall, cyclic(Val-Pro), tryptophan and uracil induced a harmonious interaction between the SA- and JA-pathways to against *H. glycines*.

In summary, we found six active ISR determinants, cyclic(Pro-Tyr), cyclic(Val-Pro), cyclic(Leu-Pro), uracil, phenylalanine, and tryptophan produced by *B. simplex* Sneb545 that efficiently controlled *H. glycines*. In addition, three types of substances cyclic (Val-Pro), tryptophan and uracil were selected to treat soybeans, and the expression levels of defense-related genes were assessed using qRT-PCR. The results indicate cyclic(Val-Pro), tryptophan and uracil induced the expression of defense-related genes involved in the SA- and JA-pathways. In future studies, we will focus on the molecular and metabolic mechanisms of the six active compounds identified, as new biological control agents for the management of high glycine in soybean production.

## Materials and methods

### Bacteria and nematodes

*Bacillus simplex* Sneb545 strain was maintained in the Nematode Institute of Northern China (NINC), Shenyang Agriculture University, China^30^ and grown on lysogeny broth (LB) agar medium at 28 °C for 24 h. A single colony was inoculated to 100 mL LB liquid medium and cultivated at 28 °C for 48 h on a rotary shaker at 175 rpm for further treatments.

*Heterodera glycines* race 3 was obtained from the experimental field of the NINC. The cysts were obtained by pouring infested soil through a nested 850 μm pore over 250 μm pore sifter followed by centrifugal flotation in a 35% sucrose solution^61,62^. The cysts were surface-sterilised using 0.5% sodium hypochlorite for 3 min, rinsed with sterile distilled water seven times, and then incubated at 26 °C in the dark^14^. Second-stage juveniles (J2) were collected for SCN inoculum (1,000 J2 mL^-1^).

### Preliminary screening of ISR determinants from *Bacillus simplex* Sneb545

The supernatant and bacterial precipitate were collected by centrifuging the fermentation broth at 3,952 × *g* for 10 min. The bacterial precipitate was washed three times with sterilised water, and then centrifuged at 3,952 × *g*. The bacterial precipitate was retained and sterile water was added to obtain a bacterial suspension. Soybean seeds were coated with fermentation supernatant and bacterial suspension for ISR bioassay, and seeds coated with sterile water were used as a control.

### ISR bioassay

**Figure 14:**
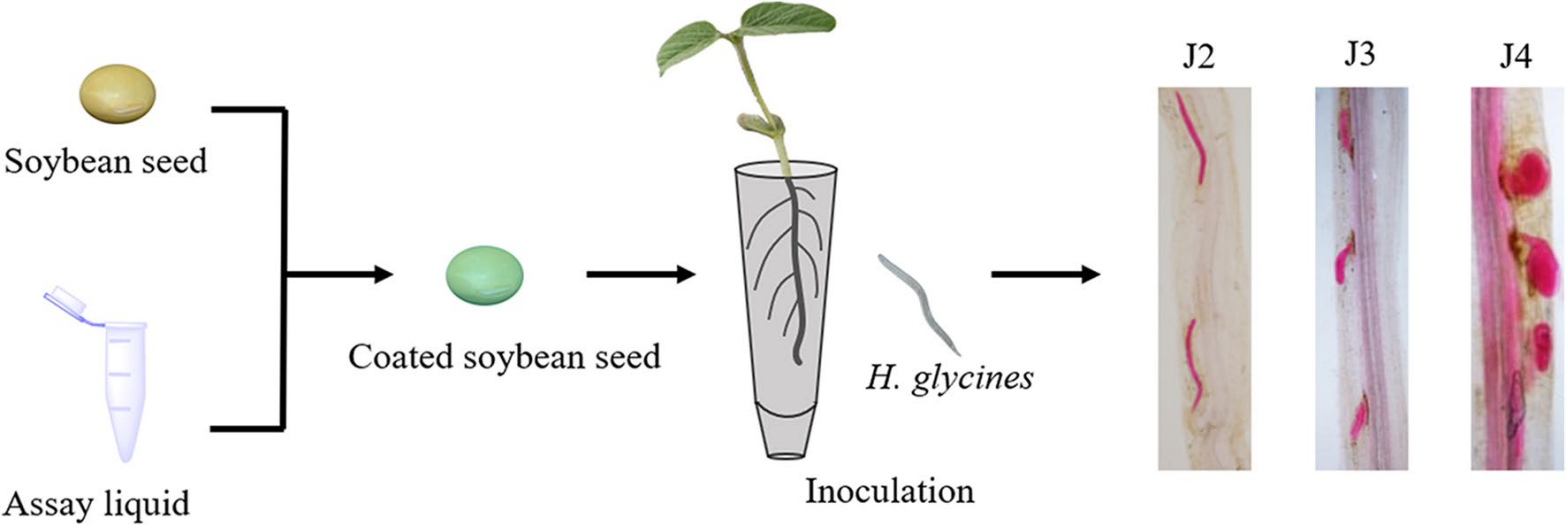
The process of ISR bioassay. The soybean seeds coated with various assay liquid at 12 dpi inoculation, and then counted the number of J2, J3 and J4 within the roots.

The susceptible soybean seeds, Liaodou 15, were surface sterilised with 0.5% sodium hypochlorite for 10 min and washed seven times with sterilised water^63^. Sterilised seeds were coated with assay liquid, at a ratio of 1:70 (mL:g), and were then air-dried at room temperature for 12 h and grown in plastic culture tubes (40 × 140 mm) containing sterilised sand^15^. At the second true leaf stage, the seedlings were inoculated with 2 ml freshly hatched J2 (approximately 1,000) by pipetting into two 1-cm-deep holes around the stem base. The roots were collected 12 d after inoculation and stained with acid fuchsin solution^64^. The number of different stage nematodes within the root were counted under a microscope (Nikon, Tokyo, Japan) (Fig. 14). ISR bioassay was evaluated using the number of nematodes within the root and the J4 ratio^65,66^.

### Extraction of ISR determinants from *Bacillus simplex* Sneb545 supernatant

To obtain Sneb545 fermentation broth, the strain was incubated with LB medium (25 L) in the fermenter at 28 °C and 220 rpm for 72 h. The fermentation broth was then separated by centrifugation at 3,952 × *g* for 10 min, and the supernatant was collected to use as a fermentation broth. The broth was concentrated to 1/10 volume by lyophilisation, and then four times the volume of ethanol was added. The concentrate was kept overnight at 4 °C, and the precipitate and supernatant were obtained by filtering. The concentrated supernatant was placed in a rotary evaporator (BUCHI I-100, Flawil, Switzerland), then the concentrates and precipitates were freeze-dried. The lyophilised powder was dissolved in 2% dimethyl sulfoxide (DMSO) to form a 20 mg/mL solution as the assay liquid for ISR bioassay, and 2%DMSO was used as a control.

The lyophilised supernatant powder was prepared in a 10% aqueous solution, and then extracted three times with PET followed by CHCl_3_, ETOAC, and nBuOH progressively. The final AP and all extracts were concentrated and then freeze-dried. The lyophilised powders were dissolved in 2% DMSO to form a 20 mg/mL solution as the assay liquid for ISR bioassay, and 2% DMSO was used as a control.

### Separation and purification of n-butanol extract

The n-butanol extract (7.5 g) was dissolved in methanol and then mixed with silica gel in a ratio of 1:1.5. When the solvent was completely evaporated, the homogeneous mixture was chromatographed on a silica gel column (60 × 50 mm ID), then progressively eluted using dichloromethane: acetone at 9:1 and 7:3; dichloromethane: methanol at 95:5, 9:1, 8:2, 7:3 and 5:5; followed by methanol: water at 8:2. Forty millilitres of eluent was collected per tube, with 110 tubes in total. Similar fractions were combined using thin-layer chromatography (TLC), and then freeze-dried. The lyophilised various powders were dissolved in 2% DMSO to form a 5 mg/mL solution as the assay liquid for ISR bioassay, and 2% DMSO was used as a control.

### Separation of S5 through semi-preparative HPLC

Fraction S5 was dissolved in methanol and filtered, then further separated and purified by semi-preparative HPLC (Waters 600E, Milford, America). Chromatographic conditions were as follows: semi-preparative AQ-C18 Column (10 × 250 mm, 10 μm, Ultimate); the mobile phase of acetonitrile: water = 15:85 (v: v); flow rate of 1.5 mL/min; temperature of 25 °C; detection wavelength of 213 nm; 100 μL per injection. The eluate was collected corresponding to the absorption peak and the purity was assessed using analytical HPLC (Agilent 1260, Palo Alto, America).

### Separation of S11 by Sephadex LH-20 column

The absorbance of fraction S11 was determined by an ultraviolet spectrophotometer (Shimadzu UV2450, Kyoto, Japan). Fraction S11 was chromatographed on a gel Sephadex LH-20 column (100 × 16 mm ID) and using methanol: water at 8:2 as the eluting agent. The flow rate was 0.3 mL/min and the detection wavelength was 220 nm. Similar fractions were combined based on UV detection peaks, and then freeze-dried. The lyophilized various powders were dissolved in 2% DMSO in a 5 mg/mL solution as the assay liquid for ISR bioassay, and 2% DMSO was used as a control.

### Separation of S11-6 by semi-preparative HPLC

Fraction S11-6 was dissolved in methanol and filtered, then further separated and purified by semi-preparative HPLC. The chromatographic conditions were similar to those described in “Separation of S5 through semi-preparative HPLC” with some modifications, such as the mobile phase of acetonitrile: water was 10:90 (v: v). The eluate was collected according to the absorption peak and the purity was assessed by analytical HPLC.

### Structural identification and ISR bioassay for active compounds

The structures of six active compounds were identified using nuclear magnetic resonance spectroscopy (Bruker 600 MHz, Karlsruhe, Germany). The nuclear magnetic conditions were as follows: the sample was dissolved in deuterated reagent and the scanned range of ^1^H NMR was from − 2 to 16 ppm and ^13^C NMR was from 0 to 220 ppm at 25 °C. The active compounds were dissolved in 2% DMSO to 1 mM solution as the assay liquid for ISR bioassay, and 2% DMSO was used as a control.

### Real-time PCR analysis of gene expression

Soybeans were treated with cyclic(Val-Pro), tryptophan and uracil, respectively, and sterilised water was used as control. Soybeans were grown as described in ISR bioassay and inoculated with 1,000 J2s of *H. glycines*. Roots were collected at 1, 5 and 10 dpi, and then were frozen in liquid nitrogen immediately and stored at –80 °C until further use. Total RNA was extracted using the RNA pure Plant Kit (Takara, China) according to the manufacturer’s instructions. Total RNA (1 μg) was converted to cDNA using the RT Reagent Kit (Takara, China). qRT-PCR was performed on the CFX Connect Real-Time PCR Detection System (Bio-Rad, USA). The genes expression of PR genes were detected, and *Actin 11* was used as reference gene for normalizing the target genes. The primers for *PR1*^67^, *PR2*^68^, *PR3a*^58^, *PR3b*^58^, *PR10*^59^ and *Actin 11*^14^ were described in Table 1. The 10-μl reaction mixtures of qRT-PCR contained 5 μL 2 × TB Green premix (Takara), 1 μL cDNA template (1:5 dilution), 3.2 μL sterile distilled water, and 0.8 μL of each forward and reverse primers for selected genes. The reaction conditions were 95 °C for 30 s, followed by 40 cycles at 95 °C for 5 s and 60 °C for 30 s. A melting curve analysis was performed after 40 cycles to confirm that a single product was present for each reaction. There were three biological replicates and three technical replicates for each treatment per time-point, and the data were quantified using the 2^-ΔΔCt^ method^69^.

**Table 1:** PCR primers of selected PR genes used for qRT-PCR amplification.

Gene	Forward/reverse primers 5′–3'
*Actin11*	F: CGGTGGTTCTATCTTGGCATC
*Actin11*	R: GTCTTTCGCTTCAATAACCCTA
*PR1*	F:CTCACCAACAGACTATGTTAATGC
*PR1*	R: CGAGTTTGCAGTCACCTTTG
*PR2*	F: GTTCGGAATGTGAAGCAAGGA
*PR2*	R: ATAGGAGAAAAGAGCCGCCAA
*PR3a*	F: AGCAGGGTCCTTGTTATTCG
*PR3a*	R: TGCCAGCACAGCCAGAGT
*PR3b*	F: AAACCCCACAACTCAACCTT
*PR3b*	R: GTCACTCCCACCAGACTCAAT
*PR10*	F: GCTTGCGGGTGACAAATAC
*PR10*	R: ACACTCCCACGTCCAAATC

### Statistical analysis

All data were analysed using SPSS Statistics 22 (SPSS Inc., IL, USA). The differences among the means were calculated using ANOVA models. Significant differences between different developmental stages for soybean cysts in roots were determined according to Duncan’s multiple range test (*P* < 0.05). Significant differences between treatments in the defense gene expression analysis experiments were evaluated using t-tests (*P* < 0.05).

## Data Availability

All the data supported the findings of this study are available from the corresponding author by request.
